# Supplementary material for: Nanoparticle-Induced Augmentation of Neutrophils’ Phagocytosis of Bacteria
Source: Front Pharmacol. 2022 Jul 4;13:923814. doi: 10.3389/fphar.2022.923814 (PMC9289463; doi:10.3389/fphar.2022.923814)

## Slide 1
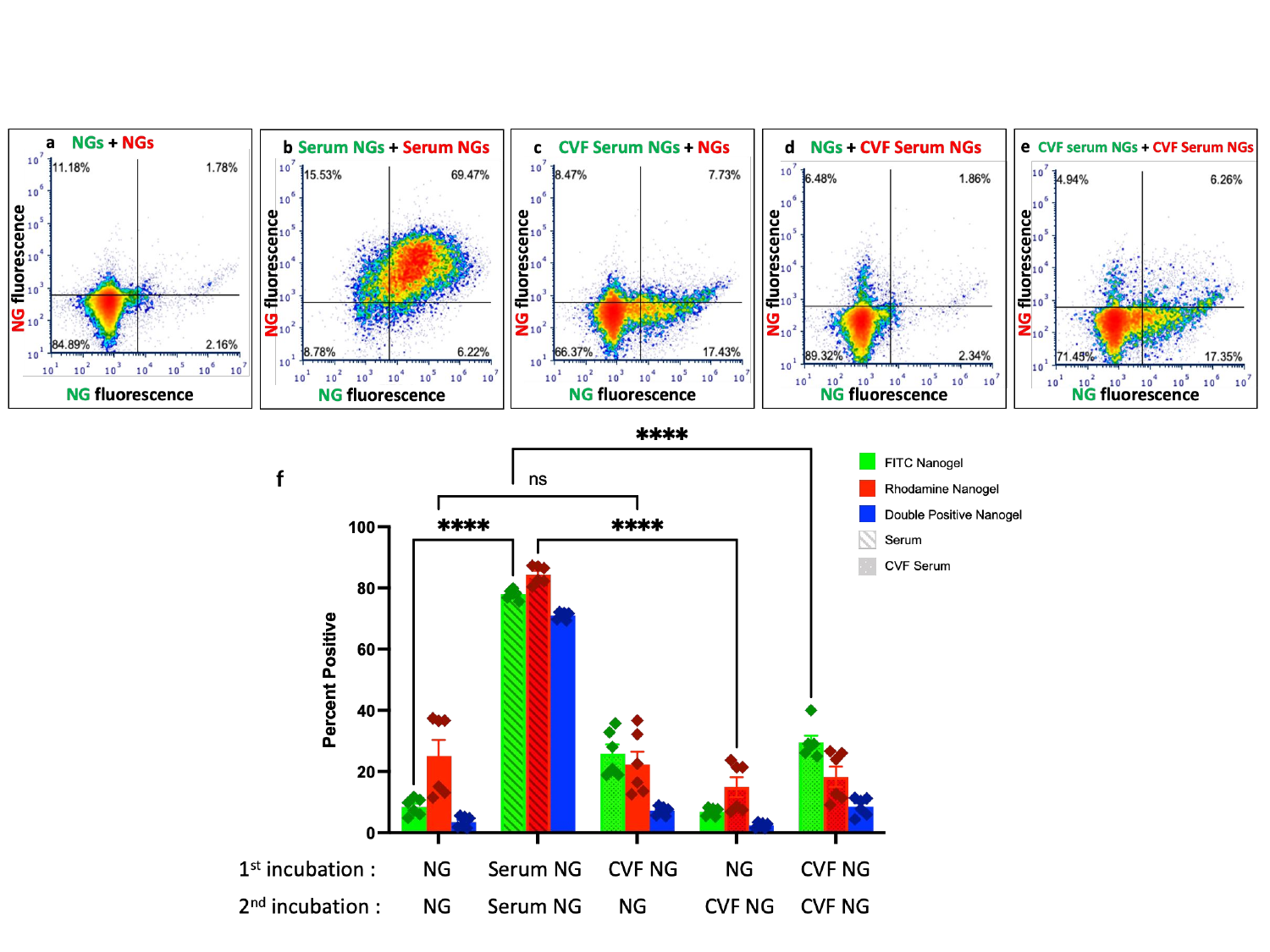

f

## Slide 2
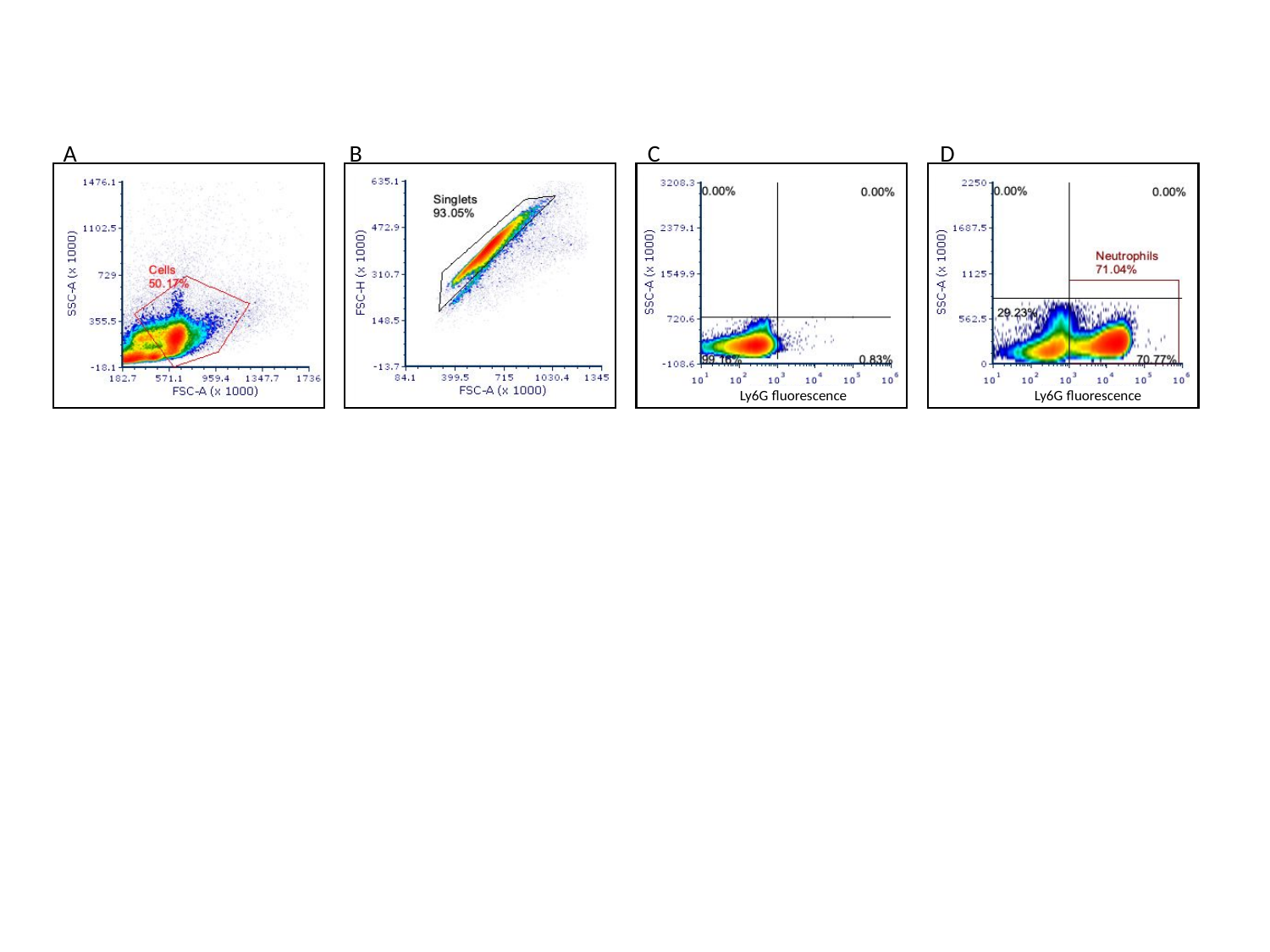

A
B
C
D
Ly6G fluorescence
Ly6G fluorescence

## Slide 3
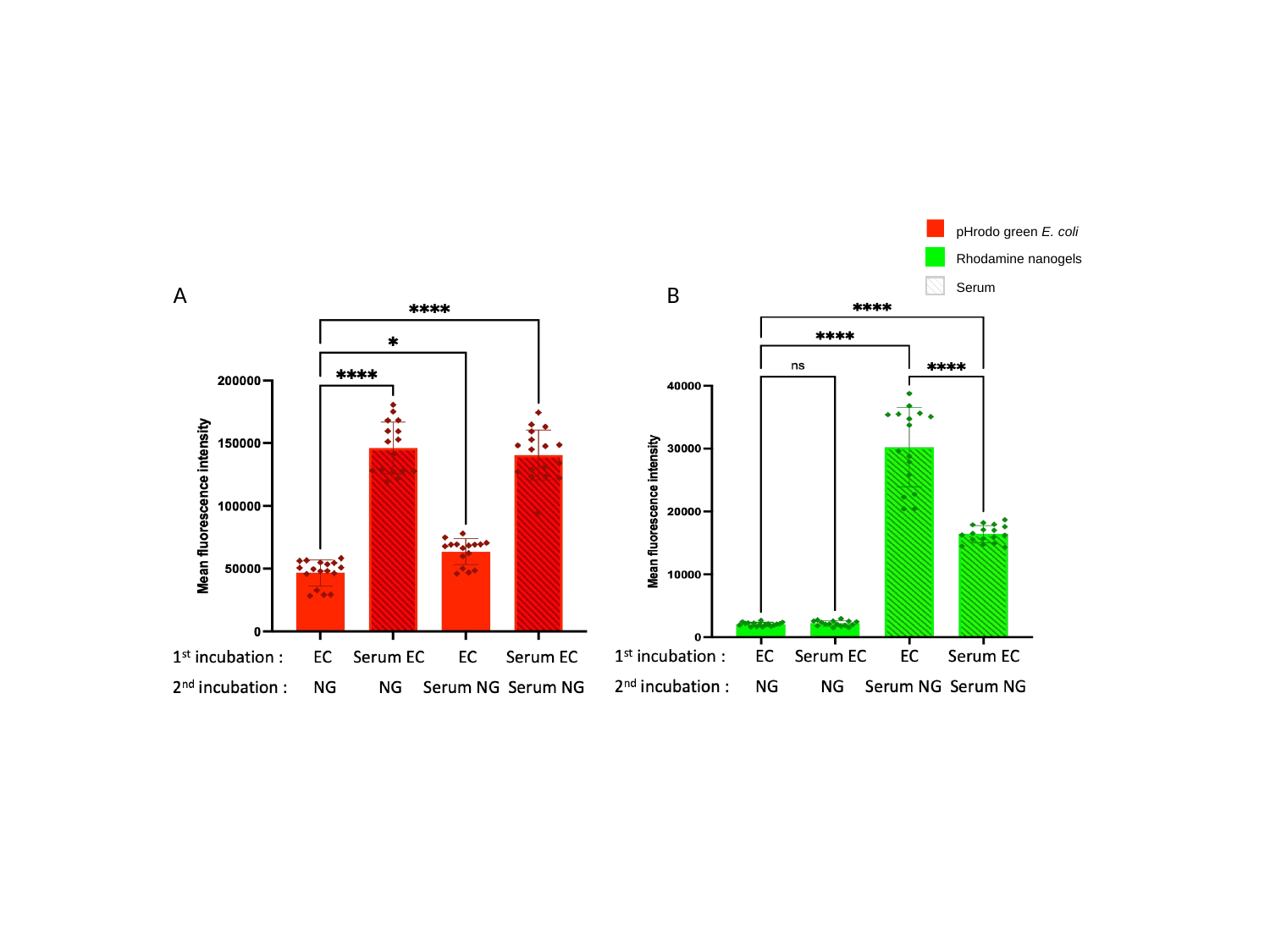

pHrodo green E. coli
Rhodamine nanogels
Serum
A
B

## Slide 4
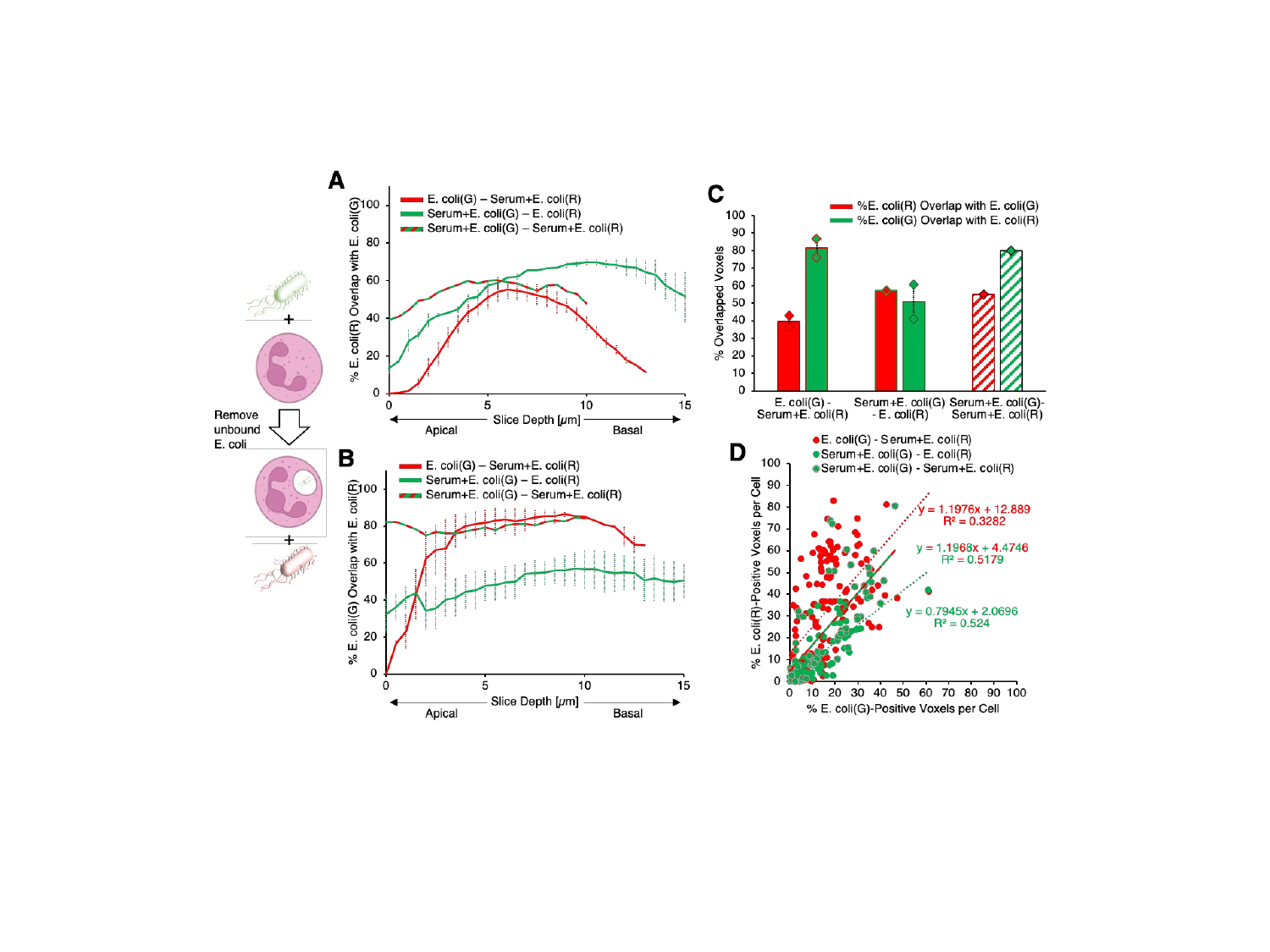

## Slide 5
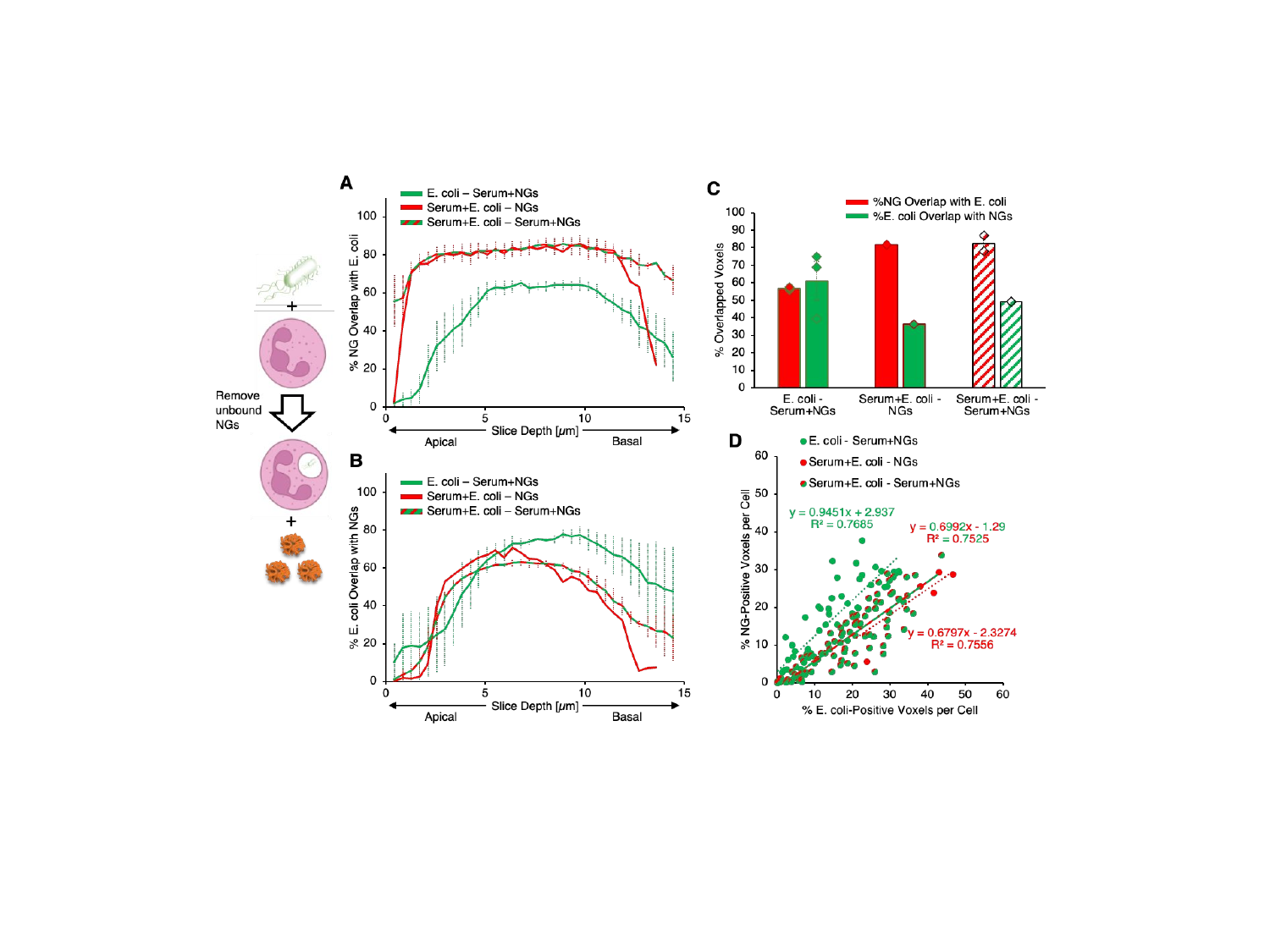

## Slide 6
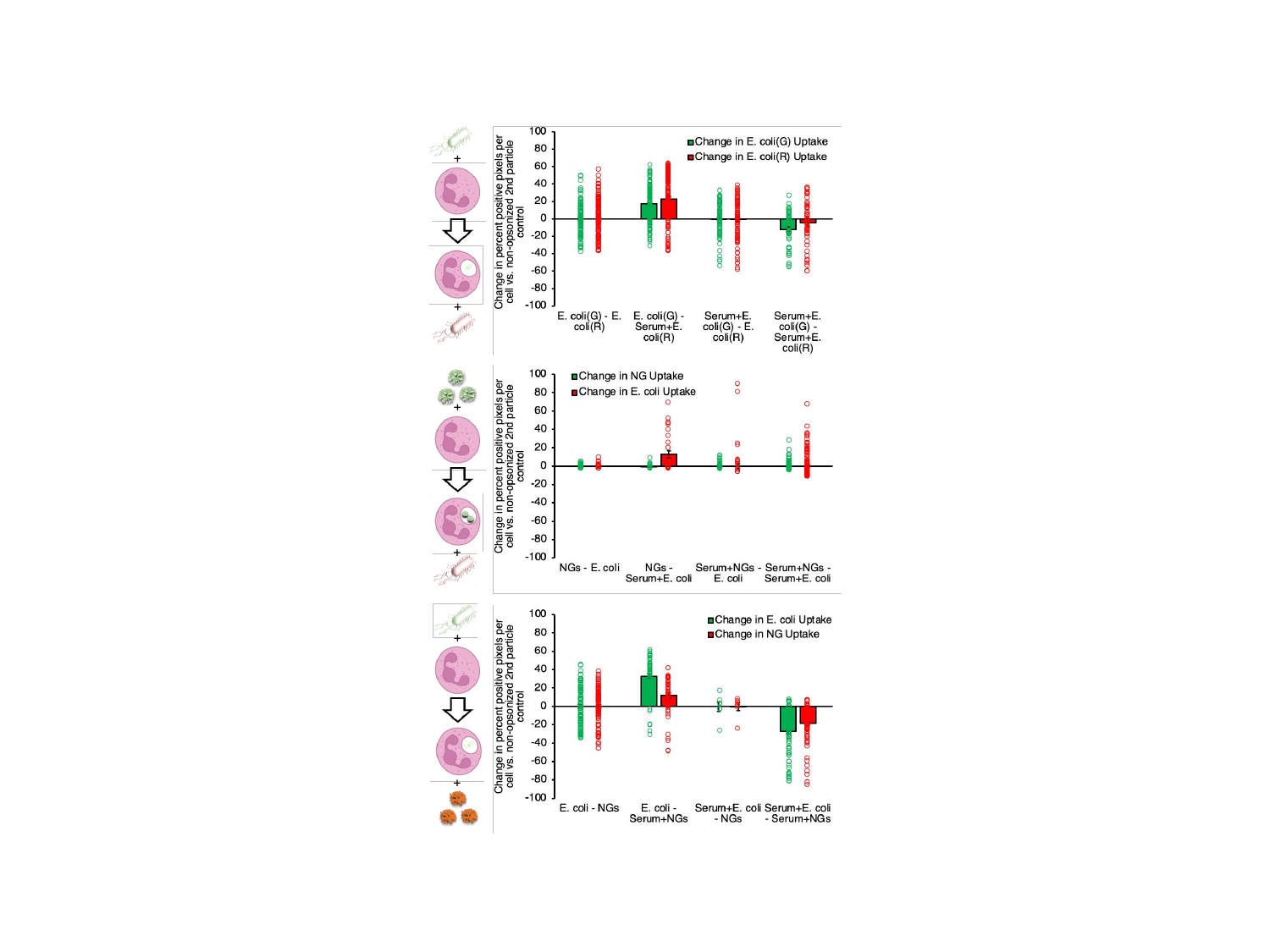

## Slide 7
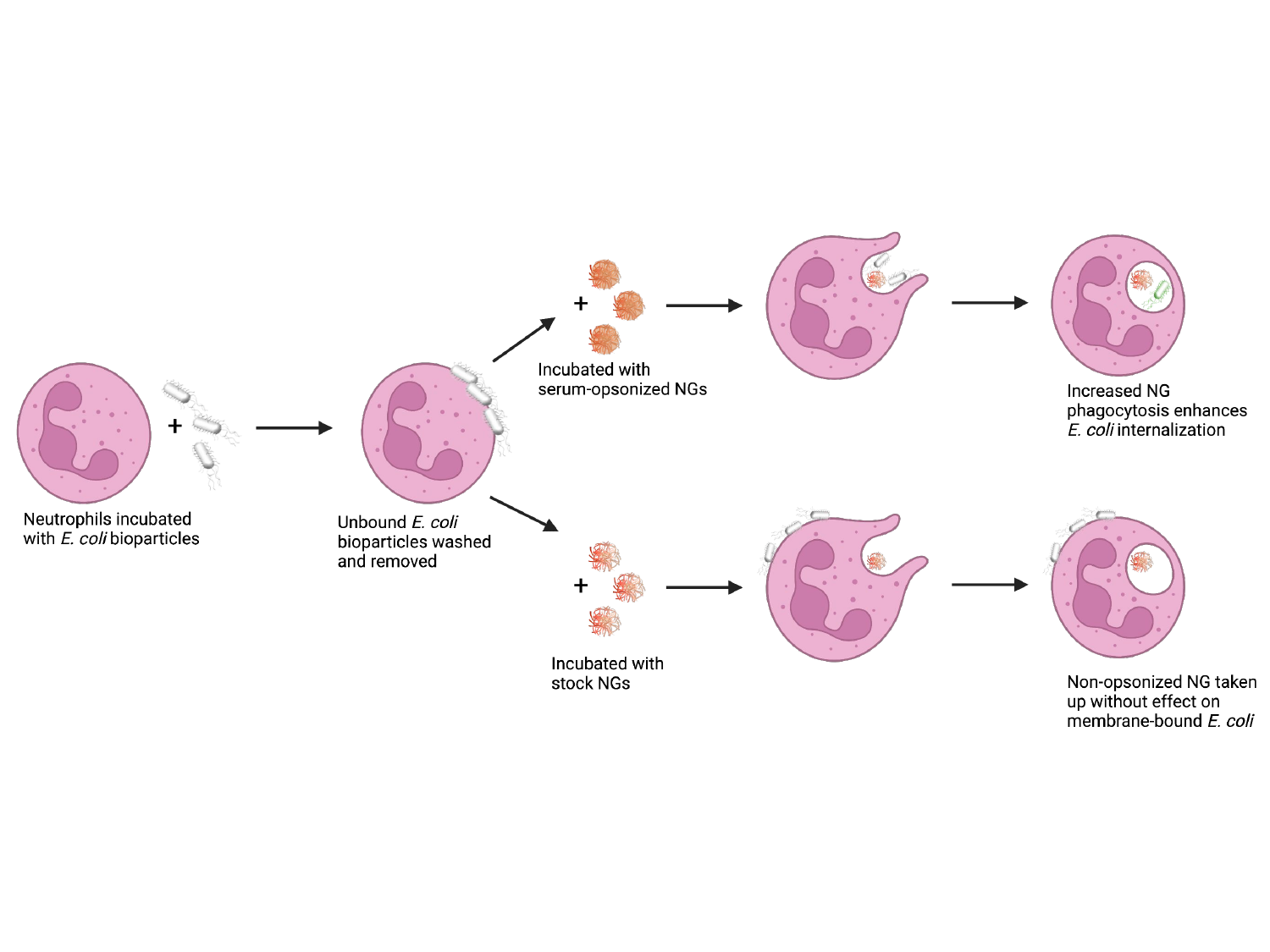

Supplement: Supplementary file 3 [file Presentation1.PPTX]
