# Supplementary material for: Nanoparticle-Induced Augmentation of Neutrophils’ Phagocytosis of Bacteria
Source: Front Pharmacol. 2022 Jul 4;13:923814. doi: 10.3389/fphar.2022.923814 (PMC9289463; doi:10.3389/fphar.2022.923814)
Supplement: Supplementary file 8 [file DataSheet1.DOCX]

//Script 1: Taking initial TIFs and saving as single channel stacks

//You need to run this for all images you want to analyze, before running any of the other scripts

//TIFs were extracted from .lif file stacks and saved in folders with the same name as the TIF

//You will be prompted to enter the name of the TIF image you want to process - Enter the name without the extension

//Below, define filepathw with the directory containing the images you want to analyze

filepathw = "/directorywithimages/";

catname = "Image Name";

Dialog.create("Choose image to process");

Dialog.addString("Image Name:", catname);

Dialog.show();

catname = Dialog.getString();

open(filepathw+catname+"/"+catname+".tif");

selectWindow(catname+".tif");

//Splitting the TIF of the confocal stack into individual color channels - You may need to rearrange the channel names. Here, C4=Membrane stain, C3=Red fluorescence, C2=Green fluorescence, C1=DAPI

run("Split Channels");

selectWindow("C4-"+catname+".tif");

saveAs("Tiff", filepathw+catname+"/"+catname+"_Membrane.tif");

selectWindow("C3-"+catname+".tif");

saveAs("Tiff", filepathw+catname+"/"+catname+"_Red.tif");

selectWindow("C2-"+catname+".tif");

saveAs("Tiff", filepathw+catname+"/"+catname+"_Green.tif");

selectWindow("C1-"+catname+".tif");

saveAs("Tiff", filepathw+catname+"/"+catname+"_DAPI.tif");

close("*");

//Script 2: Saving individual slices and stacks from each color channel as thresholded mask images

//Run this before running Script 3, for all channels you want to compare via threshold colocalization analysis

//You will be prompted to enter the name of the image and stain you want to process, along with the lower threshold defining positive vs. negative staining

//Here, thresholds were determined empirically as DAPI=32, Red=12, Green=16, Membrane=16, based on Renyi filtering of a few sample image slices.

//Below, define filepathw with the directory containing the images you want to analyze

filepathw = "/directorywithimages/";

catname = "Image Name";

stain = "Stain";

Dialog.create("Choose file and stain to process");

Dialog.addString("Image Name:", catname);

Dialog.addString("Stain:", stain);

Dialog.addNumber("Lower Threshold:", stainthresh);

Dialog.show();

catname = Dialog.getString();

stain = Dialog.getString();

stainthresh = Dialog.getNumber();

open(filepathw+catname+"/"+catname+"_"+stain+".tif");

selectWindow(catname+"_"+stain+".tif");

bigindex=nSlices+1;

//Despeckle can be eliminated if desired, but it was used here to generate thresholded images that indicate areas in the image with more blocks of signal

run("Despeckle", "stack");

run("Stack to Images");

for (i=1; i<bigindex; i++)

{

nameindex=d2s(i,0);

//If there are more than 100 slices in the stack, add a third conditional below, with i>=100

if (i<10)

{name = catname+"_"+stain+"-000"+nameindex;}

if (i>=10)

{name = catname+"_"+stain+"-00"+nameindex;}

selectWindow(name);

setTheshold(stainthresh, 255);

//Optional lines below for hard-coding the stain thresholds

// if (stain=="DAPI")

// {setThreshold(32, 255);}

// if (stain=="Red")

// {setThreshold(12, 255);}

// if (stain=="Green")

// {setThreshold(16, 255);}

// if (stain=="Membrane")

// {setThreshold(16, 255);}

setOption("BlackBackground", false);

run("Convert to Mask");

saveAs("Tiff", filepathw+catname+"/"+stain+"/"+name+".tif");

}

close("*");

for (i=1; i<bigindex; i++)

{

nameindex=d2s(i,0);

//If there are more than 100 slices in the stack, add a third conditional below, with i>=100

if (i<10)

{name = catname+"_"+stain+"-000"+nameindex;}

if(i>=10)

{name = catname+"_"+stain+"-00"+nameindex;}

open(filepathw+catname+"/"+stain+"/"+name+".tif");

}

run("Images to Stack", "name="+stain+"_Thresh title=[] use");

saveAs("Tiff", filepathw+catname+"/"+catname+"_"+stain+"_Thresh.tif");

close("*");

//Script 3: Analysis of overlap between thresholded images

//You will be prompted to enter the name of the image and stains you want to compare. Make sure you've used Script 2 to save thresholded images for those stains.

//Below, define filepathw with the directory containing the images you want to analyze

filepathw = "/directorywithimages/";

catname = "Image Name";

stain1 = "Stain 1";

stain2 = "Stain 2";

Dialog.create("Choose file and stains to process");

Dialog.addString("Image Name:", catname);

Dialog.addString("Stain 1:", stain1);

Dialog.addString("Stain 2:", stain2);

Dialog.show();

catname = Dialog.getString();

stain1 = Dialog.getString();

stain2 = Dialog.getString();

open(filepathw+catname+"/"+catname+"_"+stain1+"_Thresh.tif");

open(filepathw+catname+"/"+catname+"_"+stain2+"_Thresh.tif");

selectWindow(catname+"_"+stain1+"_Thresh.tif");

bigindex=nSlices+1;

//Creating an image representing the intersection of the two thresholded image stacks

imageCalculator("AND create stack", catname+"_"+stain1+"_Thresh.tif",catname+"_"+stain2+"_Thresh.tif");

selectWindow("Result of "+catname+"_"+stain1+"_Thresh.tif");

for (i=1; i<bigindex; i++)

{

run("Measure");

run("Next Slice [>]");

}

selectWindow(catname+"_"+stain1+"_Thresh.tif");

for (i=1; i<bigindex; i++)

{

run("Measure");

run("Next Slice [>]");

}

selectWindow(catname+"_"+stain2+"_Thresh.tif");

for (i=1; i<bigindex; i++)

{

run("Measure");

run("Next Slice [>]");

}

//Measurement outputs will be blocks of measurements with; 1) Intersection images; 2) Stain 1 images; 3) Stain 2 images for all slices in the stacks

close("*");

//Script 4: Cell ROI analysis

//For this analysis, we manually drew ROIs for each cell based on the membrane stains

//The ROIs were all collected in ImageJ's ROI manager and ROI collections for each image were saved as .zip files, named identically to the title of the image

//You will be prompted to enter the name of the image and stains you want to compare. Make sure you've saved thresholded images for those stains (Script 2)

//You will also be prompted to enter the number of cell ROIs in the image. You'll need to note the number of ROIs you drew for your image.

filepathw = "/Users/spraguecleghornx/Desktop/Session 1 Jia (10-18-2021, SG+SR)/10_28/";

catname = "Image Name";

stain1 = "Stain 1";

stain2 = "Stain 2";

roiindex = 1;

Dialog.create("Choose file and stains to process");

Dialog.addString("Image Name:", catname);

Dialog.addString("Stain 1:", stain1);

Dialog.addString("Stain 2:", stain2);

Dialog.addNumber("# of ROIs:", roiindex);

Dialog.show();

catname = Dialog.getString();

stain1 = Dialog.getString();

stain2 = Dialog.getString();

roiindex = Dialog.getNumber();

open(filepathw+catname+"/"+catname+"_"+stain1+".tif");

selectWindow(catname+"_"+stain1+".tif");

bigindex=nSlices+1;

roiManager("Open", filepathw+catname+"/"+catname+".zip");

close("*");

for (i=1; i<bigindex; i++)

{

//If there are more than 100 slices in your stack, add a third conditional below, with i>=100

nameindex=d2s(i,0);

if (i<10)

{

name1 = catname+"_"+stain1+"-000"+nameindex;

name2 = catname+"_"+stain2+"-000"+nameindex;

}

if (i>=10)

{

name1 = catname+"_"+stain1+"-00"+nameindex;

name2 = catname+"_"+stain2+"-00"+nameindex;

}

open(filepathw+catname+"/"+stain1+"/"+name1+".tif");

open(filepathw+catname+"/"+stain2+"/"+name2+".tif");

//The measurements below will output alternating lines with; 1) Quantity of red-positive voxels in the cell ROIs; 2) Quantity of green-positive voxels in the cell ROIs

for (j=0; j<roiindex; j++)

{

selectWindow(name1+".tif");

roiManager("Select",j)

run("Measure");

selectWindow(name2+".tif");

roiManager("Select",j)

run("Measure");

}

close("*");

//Total output here will be blocks of measurements for each ROI, with each ROI block covering all slices in the stack

}

//Script 4: Cell ROI analysis (***Widefield Image Version***)

//For this analysis, I manually drew ROIs for each cell based on the membrane stains

//The ROIs were all collected in ImageJ's ROI manager and ROI collections for each image were saved as .zip files, named identically to the title of the image

//You will be prompted to enter the name of the image and stains you want to compare. Make sure you've saved thresholded images for those stains

//You will also be prompted to enter the number of cell ROIs in the image. You'll need to note the number of ROIs you drew for your image.

filepathw = "/Users/spraguecleghornx/Desktop/Active2021/KMR_Images/Session 1 Jia (10-18-2021, SG+SR)/1_28/";

catname = "Image Name";

stain1 = "Stain 1";

stain2 = "Stain 2";

roiindex = 1;

Dialog.create("Choose file and stains to process");

Dialog.addString("Image Name:", catname);

Dialog.addString("Stain 1:", stain1);

Dialog.addString("Stain 2:", stain2);

Dialog.addNumber("# of ROIs:", roiindex);

Dialog.show();

catname = Dialog.getString();

stain1 = Dialog.getString();

stain2 = Dialog.getString();

roiindex = Dialog.getNumber();

open(filepathw+catname+"/"+catname+"_"+stain1+"_MIP.tif");

open(filepathw+catname+"/"+catname+"_"+stain2+"_MIP.tif");

roiManager("Open", filepathw+catname+"/"+catname+".zip");

for (j=0; j<roiindex; j++)

{

selectWindow(catname+"_"+stain1+"_MIP.tif");

roiManager("Select",j)

run("Measure");

selectWindow(catname+"_"+stain2+"_MIP.tif");

roiManager("Select",j)

run("Measure");

}

close("*");

//Total output here will be blocks of measurements for each ROI, with each ROI block covering all slices in the stack
